# Risk factors and prediction model of level II lymph node metastasis in papillary thyroid carcinoma

**DOI:** 10.3389/fonc.2022.984038

**Published:** 2022-12-20

**Authors:** Chun Huang, Daixing Hu, Yuchen Zhuang, Xinliang Su

**Affiliations:** ^1^ Department of Breast and Thyroid Surgery, The First Affiliated Hospital of Chongqing Medical University, Chongqing, China; ^2^ Department of Breast and Thyroid Surgery, The Second Affiliated Hospital of Chongqing Medical University, Chongqing, China

**Keywords:** papillary thyroid carcinoma, level II, lymph node metastasis, risk factors, prediction model

## Abstract

**Introduction:**

Surgical management of lateral lymph nodes in papillary thyroid carcinoma, especially at level II, remains controversial. This study aimed to investigate the risk factors for level II lymph node metastasis in patients with papillary thyroid carcinoma and establish a prediction model to estimate the metastatic risk.

**Materials and methods:**

A total of 768 patients with papillary thyroid carcinoma underwent thyroidectomy and central plus lateral lymph node dissection, including levels VI, II, III, and IV, at the First Affiliated Hospital of Chongqing Medical University from January 2016 to December 2018. Data on the clinicopathological characteristics were collected and analyzed. Univariate and multivariate analyses were performed to identify risk factors for level II lymph node metastasis. Subsequently, a predictive model was established based on the results of the multivariate analyses.

**Results:**

The level II lymph node metastatic rate was 34.11% with the following features: largest tumor diameter >20 mm (Odds ratio=1.629, P=0.026), located in the upper pole (Odds ratio=4.970, P<0.001), clinical lymph node-positive (clinical central lymph node-positive: Odds ratio=1.797; clinical lateral lymph node-positive: Odds ratio=1.805, P=0.008), vascular invasion (Odds ratio=6.759, P=0.012), and rate of central lymph node metastasis (Odds ratio=2.498, P<0.001). Level III lymph node metastasis (Odds ratio=2.749, P<0.001) and level IV lymph node metastasis (Odds ratio=1.732, P=0.007) were independent of level II lymph node metastasis predictors. The prediction model’s areas under the receiver operating characteristic curve were 0.815 and 0.804, based on bootstrapping validation. Level II lymph node metastasis was associated with the tumor-free survival rate of patients with papillary thyroid carcinoma (P<0.001).

**Conclusions:**

Largest tumor diameter >20 mm, located in the upper pole, clinical lymph node-positive, vascular invasion, rate of central lymph node metastasis, and levels III and IV lymph node metastases were independent level II lymph node metastasis predictors. We developed a prediction model for level II lymph node metastasis. Overall, level II lymph node metastasis dissection should be individualized according to clinicopathological data both preoperatively and intraoperatively.

## Introduction

Papillary thyroid carcinoma (PTC) is the most common endocrine malignancy worldwide and its incidence has increased rapidly in recent decades (1–3). Most PTCs are mild and indolent with an excellent prognosis. However, lymph node metastasis is common in the early stages and is associated with an increased risk of recurrence and reoperation rate (4, 5). The regional nodal basins of the central and lateral neck are the first site of lymph node metastasis in PTC. Furthermore, the most common metastatic area is the central compartment (level VI), sequentially followed by levels III, IV, II, and V (6, 7).

The surgical management of lateral lymph nodes (LLNs) in PTC remains controversial. The American Thyroid Association (ATA) and National Comprehensive Cancer Network (NCCN) guidelines disagree on prophylactic lateral lymph node dissection (pLLND) (8, 9). The British Thyroid Association (BTA) guidelines suggest performing pLLND at the discretion of the patient when the central lymph node (CLN) is involved (10). Conversely, Chinese experts recommend prophylactic central lymph node dissection (pCLND) and elective LLND according to the number and proportion of central lymph node metastasis (CLNM) along with the location, size, and pathological type of PTC. However, the level II lymph node (LN-level II) metastatic rate can reach 45% (11–13), and occult metastases may also occur at level II (14, 15). Moreover, lymph node metastasis increases the risk of recurrence and reoperation rate, and most recurrences occur when LN level II is initially positive for metastatic disease (16). Albeit, blind dissection of LN-level II may increase the operative time and complications. Thus, it is necessary to carefully and accurately identify and manage patients with LN-level II positivity. This study investigated the risk factors for LN-level II metastasis in patients with PTC and aimed to establish a predictive model to estimate the risk of metastasis.

## Materials and methods

### Patient collection

The local institutional ethics committee approved this retrospective study verbally and informed consent was waived because there was no patient interest or privacy breach in the form of disclosure of personal information. A total of 2863 patients underwent surgery for thyroid diseases at the Department of Breast and Thyroid Surgery, The First Affiliated Hospital of Chongqing Medical University, Chongqing, China, between January 2016 and December 2018. The inclusion criteria were as follows: proven PTC postoperatively and modified radical neck dissection with CLND and LLND, including levels II, III, and IV. Patients with any of the following conditions were excluded: reoperation, history of surgery for other malignant tumors, distant metastasis, adolescence, and incomplete clinical information. Ultimately, 768 eligible patients were included in this study.

### Demographic and clinicopathological data

Data on the following clinicopathological variables were collected: sex, age, size, location, ultrasound-reported LN status (US-reported LN status), multifocality, laterality, capsule invasion, trachea/muscle/vascular/nerve invasion, Hashimoto thyroiditis (HT), and lymph node metastasis. The largest lesion diameter and location were recorded as the size and location. The presence of capsule invasion and trachea/muscle/vascular/nerve invasion was recorded in preoperative ultrasonography reports or intraoperative findings.

Recurrence was defined as the local or regional disease requiring treatment 6 months after the initial standard operation and diagnosed using ultrasound-guided fine-needle aspiration cytology or reoperation. The follow-ups were in an outpatient clinic. The patients were followed up using the electronic medical record system, telephone, or text messages. All of the 768 patients were effectively followed up.

## Statistical analysis

The independent-sample *t*-test or Mann–Whitney U-test was used to compare the continuous variables. Categorical variables were compared using the Chi-square test or Fisher’s exact test. Statistical significance was set at P<0.05. Variables that showed a significant association in the univariate analysis (P<0.05) were included in the multivariable logistic regression model. Based on this model, a nomogram was developed to predict the probability of LN-level II metastasis, and the predictive ability of the nomogram was measured using the receiver operating characteristic (ROC) curve, concordance index (C-index), and calibration plot. Decision curve analysis (DCA) was performed to evaluate the clinical usefulness of the nomogram in calculating the net benefits at different threshold probabilities. The Kaplan–Meier method was used to estimate disease-free survival (DFS) rates. All statistical analyses were performed using SPSS (version 25.0; SPSS Inc., Chicago, IL, United States) and graphs were generated using R software version 4.2.0.

## Results

### Patient characteristics

A total of 768 patients were included in the analysis: 243 males and 525 females; mean age, 42.0 ± 12.7 years (range, 18–85); mean tumor size, 16.3 ± 10.0 mm (range, 3–60); mean rate of CLNM, 0.33 ± 0.28; mean rate of level III lymph node (LN-level III) metastasis, 0.16 ± 0.24; mean rate of level IV lymph node (LN-level IV) metastasis, 0.10 ± 0.19. Patient characteristics are summarized in **Table 1**. Overall, the LN-level II metastasis rate was 34.11%. In the univariate analyses, we found significant differences in size, location, US-reported LN status, trachea/vascular/nerve invasion, laterality, CLNM, LN-level III metastasis, and LN-level IV metastasis between the LN-level II positive and negative groups. No significant differences were observed in sex (P=0.404), age (P=0.532), capsule invasion (P=0.168), multifocality (P=0.057), muscle invasion (P=0.116), or HT (P=0.301).

**Table 1:** Comparisons of clinicopathological factors between the negative and positive of LN-level II.

Characteristics	Total N=768	LN-level II metastasis		P-value
		Negative (n=506)	Positive (n=262)	
Sex				0.404
Male	243	155 (63.8%)	88 (36.2%)	
Female	525	351 (66.9%)	174 (33.1%)	
Age (mean ± SD, years)		42.7 ± 12.6	40.8 ± 12.9	0.050
≤ 55	663	434 (65.5%)	229 (34.5%)	0.532
>55	105	72 (68.6%)	33 (31.4%)	
Size (mean ± SD, mm)		15.2 ± 9.3	18.4 ± 10.8	<0.001
≤20	592	411 (69.4%)	181 (30.6%)	<0.001
>20	176	95 (54.0)	81 (46.0%)	
Location				<0.001
Upper	228	106 (46.5%)	122 (53.5%)	
Middle	378	276 (73.0%)	102 (27.0%)	
Low	143	110 (76.9%)	33 (23.1%)	
Isthmus	19	14 (73.7%)	5 (26.3)	
US-reported LN status				<0.001
cN0	430	326 (75.8%)	104 (24.2%)	
cN1a	91	59 (64.8%)	32 (35.2%)	
cN1b	247	121 (49.0%)	126 (51.0%)	
Multifocality				0.057
No	563	382 (67.9%)	181 (32.1%)	
Yes	205	124 (60.5%)	81 (39.5%)	
Laterality				0.002
Unilateral	587	404 (68.8%)	183 (31.2%)	
Bilateral	181	102 (56.4%)	79 (43.6%)	
Capsule invasion				0.168
No	560	377 (67.3%)	183 (32.7%)	
Yes	208	129 (62.0%)	79 (38.0%)	
Trachea invasion				0.010
No	733	490 (66.8%)	243 (33.2%)	
Yes	35	16 (45.7%)	19 (54.3%)	
Muscle invasion				0.116
No	619	416 (67.2%)	203 (32.8%)	
Yes	149	90 (60.4%)	59 (39.6%)	
Vascular invasion				0.002
No	755	503 (66.6%)	252 (33.4%)	
Yes	13	3 (23.1%)	10 (76.9%)	
Nerve invasion				0.003
No	710	478 (67.3%)	232 (32.7%)	
Yes	58	28 (48.3%)	30 (51.7%)	
HT				0.301
No	593	385 (64.9%)	208 (35.1%)	
Yes	175	121 (69.1%)	54 (30.9%)	
CLNM				<0.001
No	145	121 (83.4%)	24 (16.6%)	
Yes	623	385 (61.8%)	238 (38.2%)	
LN-level III metastasis				<0.001
No	402	331 (82.3%)	71 (17.7%)	
Yes	366	175 (47.8%)	191 (52.2%)	
LN-level IV metastasis				<0.001
No	502	376 (74.9%)	126 (25.1%)	
Yes	266	130 (48.9%)	136 (51.1%)	

LN, lymph node; US, ultrasound; cN0, clinical lymph node-negative; cN1a, clinical central lymph node-positive; cN1b, clinical lateral lymph node-positive; HT, Hashimoto thyroiditis; CLNM, central lymph node metastasis.

The ROC curve was used to analyze the diagnostic efficacy of the CLNM rate, LN level III metastasis, and LN level IV metastasis; the cut-off values are listed in **Table 2**.

**Table 2:** The cut-off values of the Rate of different levels of LNM to predict LN-level II metastasis.

Variables	AUC	Sensitivity	Specificity	P	Cut-off values
Rate of CLNM	0.685	0.531	0.743	0.020	0.4096
Rate of LN-level III metastasis	0.720	0.725	0.672	0.020	0.0607
Rate of LN-level IV metastasis	0.645	0.511	0.751	0.022	0.0460

CLNM, central lymph node metastasis; LN, lymph node.

In the multivariate analyses, size (P=0.026), location (P<0.001), US-reported LN status (P=0.008), vascular invasion (P=0.012), rate of CLNM (P<0.001), LN-level III metastasis (P<0.001), and LN level IV metastasis (P=0.007) were independent predictors of LN-level II metastasis (**Table 3**).

**Table 3:** Multivariate logistic regression analysis of factors between the negative and positive of LN-level II.

Characteristics	OR (95%CI)	P
Size		0.026
≤20	1 (reference)	
>20	1.629 (1.059-2.505)	
Location		<0.001
Middle	1 (reference)	
Upper	4.970 (3.249-7.603)	
Low	0.800 (0.473-1.353)	
Isthmus	0.803 (0.238-2.708)	
US-reported LN status		0.008
cN0	1 (reference)	
cN1a	1.797 (1.018-3.175)	
cN1b	1.805 (1.198-2.719)	
Laterality		0.101
Unilateral	1 (reference)	
Bilateral	1.416 (0.934-2.145)	
Trachea invasion No	1 (reference)	
Yes	1.188 (0.469-3.012)	0.717
Vascular invasion		0.012
No	1 (reference)	
Yes	6.759 (1.518-30.092)	
Nerve invasion		0.858
No	1 (reference)	
Yes	0.932 (0.432-2.013)	
Rate of CLNM		<0.001
≤0.4096	1 (reference)	
>0.4096	2.498 (1.680-3.716)	
Rate of LN-level III metastasis		<0.001
≤0.0607	1 (reference)	
>0.0607	2.749 (1.858-4.068)	
Rate of LN-level IV metastasis		0.007
≤0.0460	1 (reference)	
>0.0460	1.732 (1.161-2.583)	

LN, lymph node; US, ultrasound; cN0, clinical lymph node-negative; cN1a, clinical central lymph node-positive; cN1b, clinical lateral lymph node-positive; CLNM, central lymph node metastasis.

### Prediction models

**Figure 1:**
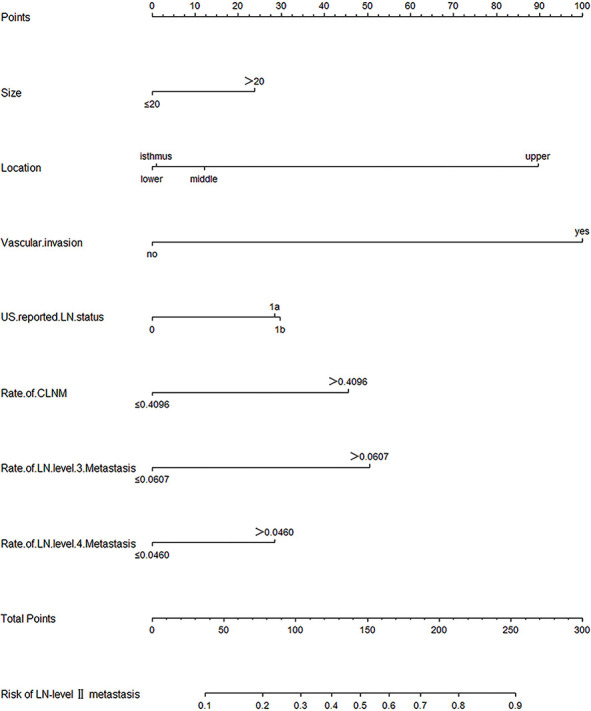
Nomogram for prediction of LN-level II metastasis. A line is drawn straight up to the point axis that corresponds with each patient variable to obtain the points. The sum of these points is located on the total score points axis. A line is drawn downwards to the risk axis to determine the possibility of LN-level II metastasis.

**Figure 2:**
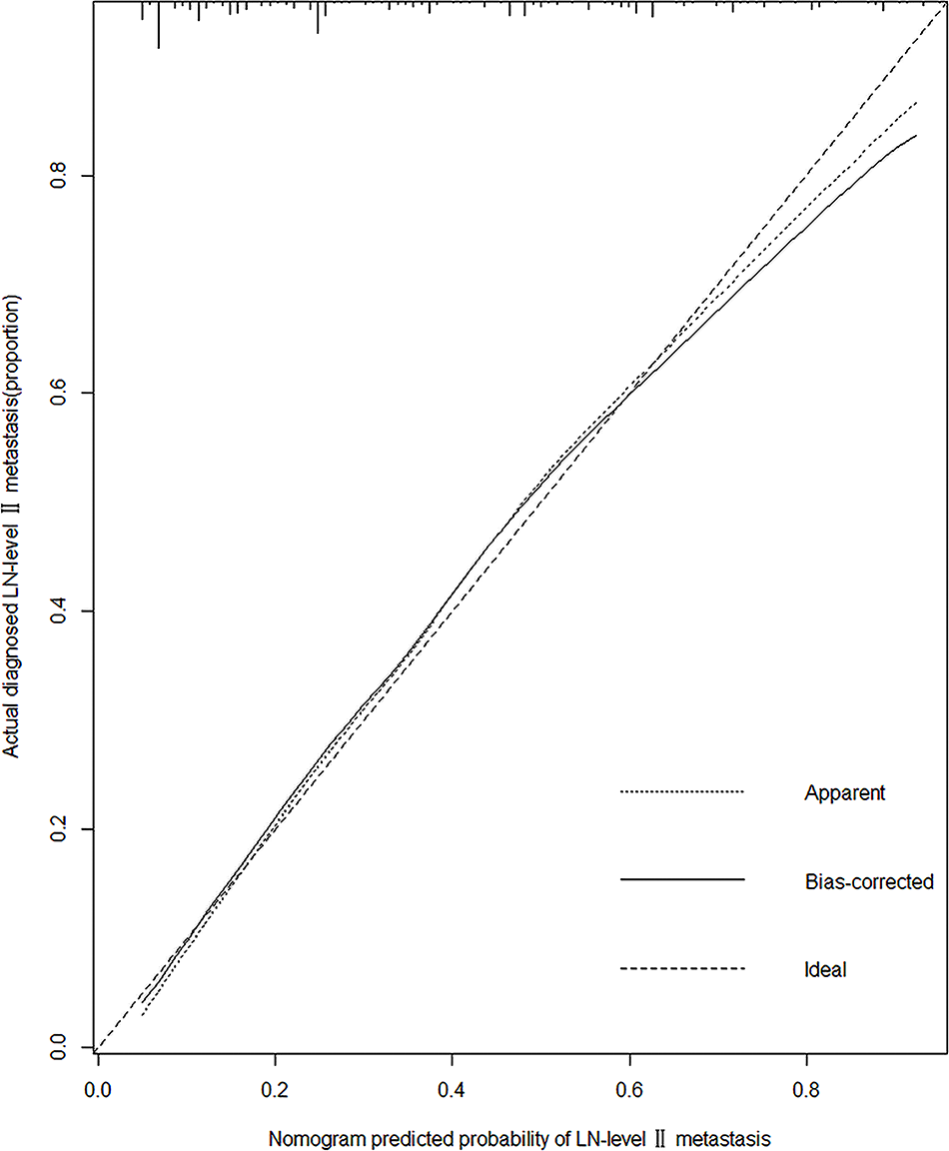
Calibration plot of the model. The calibration curve depicts the calibration of the model in terms of the agreement between the predicted risks of LN-level II metastasis and observed outcomes of LN-level II metastasis. The x-axis represents the predicted LN-level II metastasis risk. The y-axis represents the actual LN-level II metastasis rate. The diagonal dotted black line represents an ideal calibration model in which the predicted probabilities are identical to the actual outcomes. The dotted line represents the predictive performance of the nomogram; the closer the fit of the dotted line to the ideal line, the better the prediction. The black line represents the bias-corrected.

**Figure 3:**
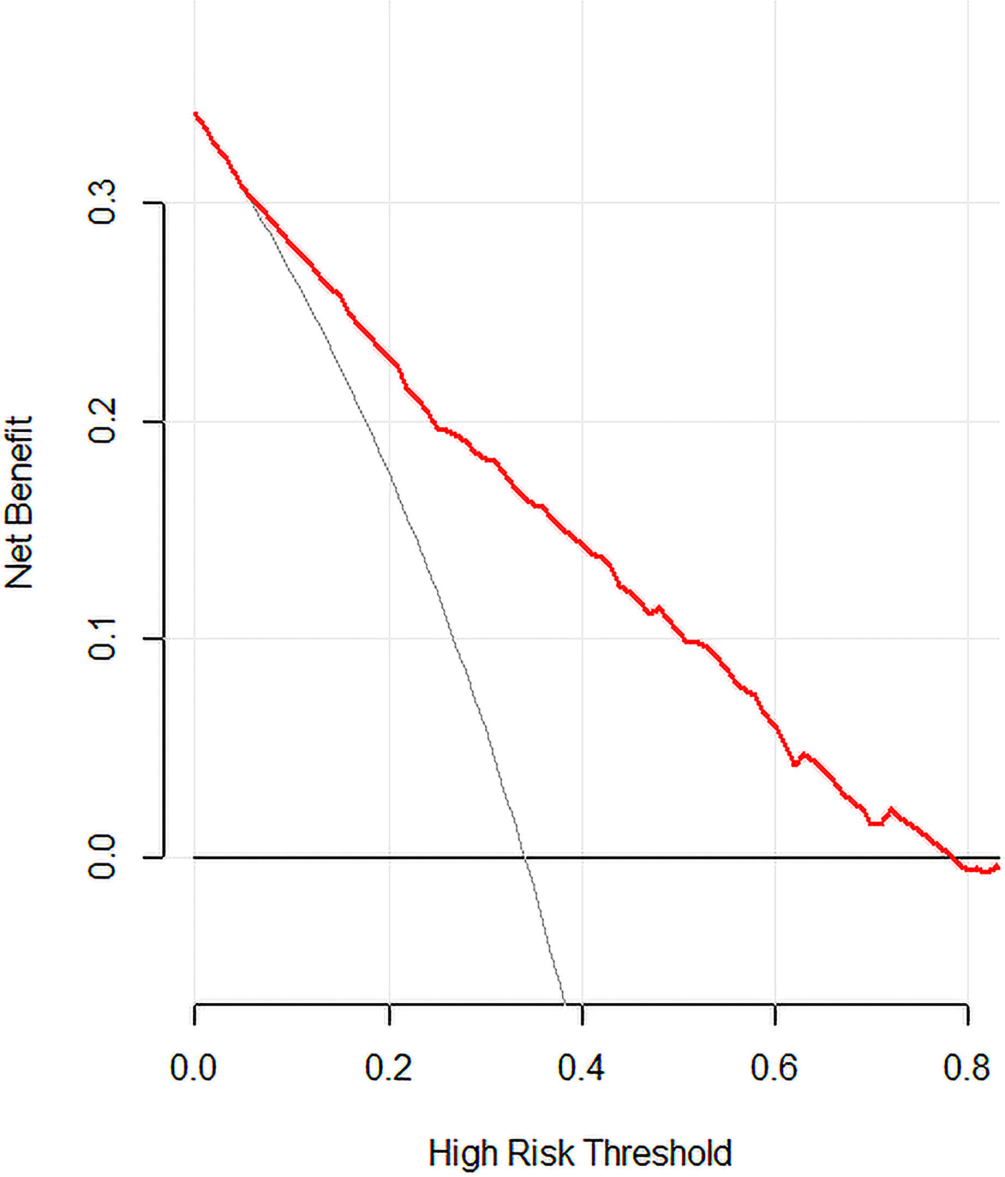
Decision curve analysis for the model. The y-axis measures the net benefit. The x-axis represents the threshold probability. The red solid line represents the nomogram. The gray line represents the hypothesis that all patients had LN-level II metastasis. The black solid line represents the hypothesis that none of the patients had LN-level II metastasis. The decision curve indicates that when the threshold probability is >10.0%, use of this predictive model would accrue greater benefit than that accruing from a treat-all or treat-none strategy.

A predictive model for LN-level II metastasis was developed based on the multivariate logistic regression analysis. A nomogram incorporating the above seven predictors is shown in **Figure 1**. The C-index for the nomogram was 0.815 (95% CI, 0.784–0.846) and 0.804 by bootstrapping validation, which demonstrated good discriminative ability. The calibration plot revealed excellent agreement between the predicted and actual observations (**Figure 2**). These results showed that the nomogram had good efficacy in predicting the probability of LN-level II metastasis. The DCA results suggested that when the threshold probability was approximately 10–90%, compared to a “treat all” or “treat none” strategy, this nomogram yielded a greater net benefit, which indicated that it had good clinical value (**Figure 3**).

### Prognostic analysis

**Figure 4:**
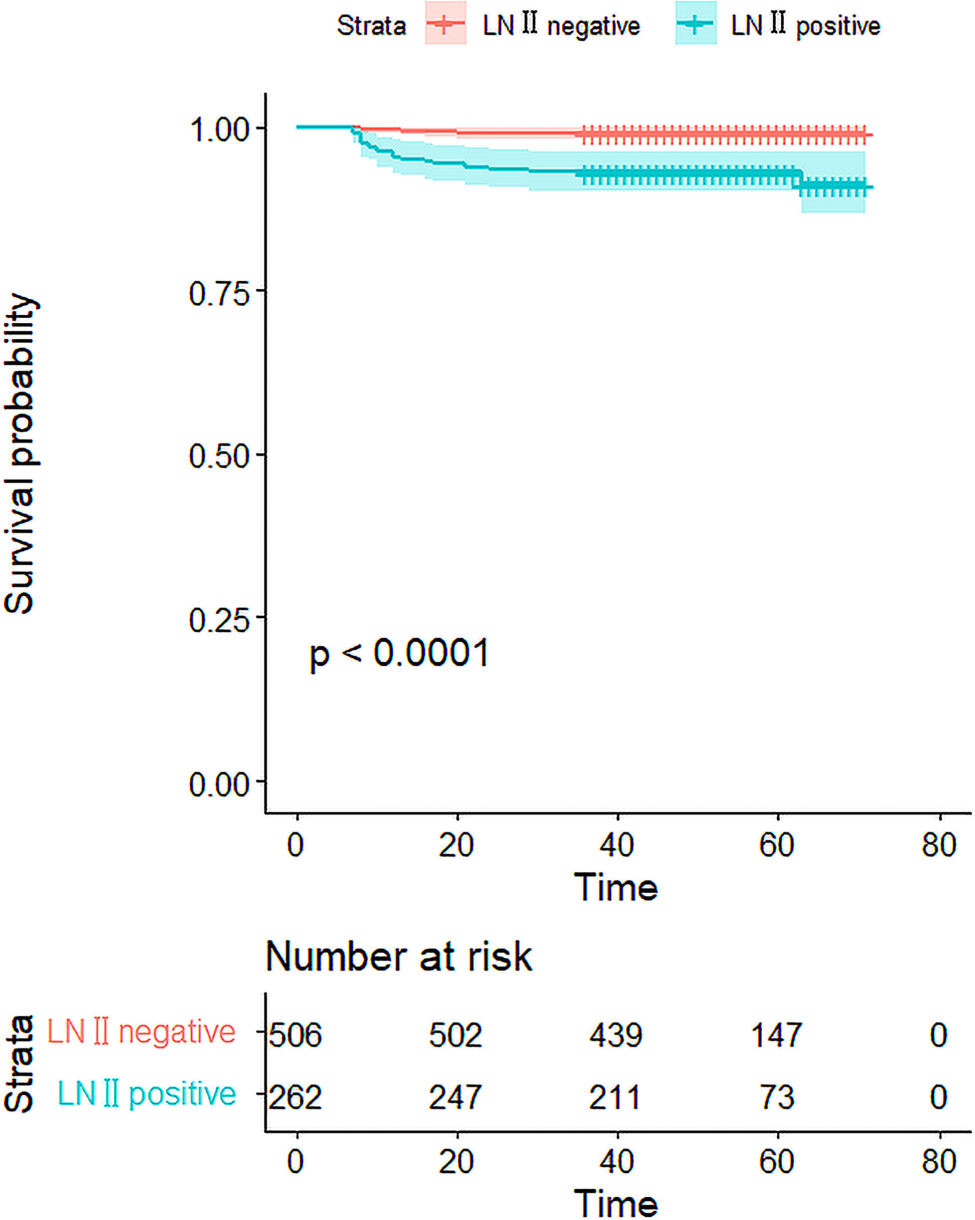
The Kaplan–Meier curves for the 768 patients. The curves show that the DFS rate was 96.9% in 768 patients, and there was a significant difference in the DFS rate between the LN-level negative and positive cohorts(p<0.001).

We investigated whether LN-level II metastasis affected recurrence. Patients were followed-up after the initial surgery until December 2021. The mean follow-up time was 51.5 ± 12.0 months (7–71 months). Among the 768 patients, 24 experienced recurrences, only five of whom were in the LN-level II negative group. Tumor-free survival of the two groups was compared and the prognosis in patients with LN-level II metastasis was worse than that of LN-level II negative (P<0.001) (**Figure 4**).

## Discussion

The present study indicated that a tumor diameter of >20 mm, tumor located in the upper pole, clinical lymph node-positivity (cN1), vascular invasion, the rate of CLNM, and LN-levels III and IV metastasis, were all positively associated with the risk of LN-level II metastasis for PTC. However, sex, age, capsule invasion, multifocality, muscle/trachea/nerve invasion, laterality, and HT were not.

Several studies have reported the probability risk factors of LN-level II metastasis for PTC, and most have reported the factors of lateral lymph node metastasis. The present study has several advantages over previous studies. First, the predictors for LN-level II metastasis in recent years were evaluated in a large cohort of patients with PTC (overall 768). Second, we calculated the relationship between the LNM rate in each compartment and LN-level II metastasis, which is more objective than the number of LNM, considering the total number of dissected LNs and the number of metastatic LNs. Third, based on the seven risk factors available in preoperative clinicopathological features and intraoperative frozen analysis, we built a predictive model and nomogram, which is a reliable tool with high accuracy for predicting LN-level II metastasis for clinicians.

Many studies have shown that a maximum tumor diameter >2 cm is associated with lateral lymph node metastasis (LLNM) in PTC and size reflects solid tumor staging (17, 18). In this study, in cases of microcarcinoma, LN-level II metastasis was detected in 25.6%. Moreover, the risk of LN-level II metastasis increased when the diameter was >2 cm (odds ratio [OR] =1.629).

Previous studies have shown that PTC located in the upper pole is more prone to lateral lymph nodes and skip metastases (19, 20). Lee reported that all cases of skip metastases occurred in patients with “upper” thyroid tumors (21). In our study, 52 out of 675 patients had skip metastasis (52/675, 7.70%) and nine cases occurred only in level II. Among them, eight were located on the upper pole with one on the middle pole. The nomogram shows that the risk of LN-level II metastasis significantly increased when the tumor was located in the upper pole. According to Qubain et al. (22), different thyroid gland locations have different lymphatic drainage pathways. Lymphatic drainage occurs in the upper direction when the tumors are located in the upper third. Furthermore, the lymphatic drainage of the lower third or isthmus is primarily collected by the lymphatic vessels accompanying the inferior thyroid artery and flows into the lateral lymph nodes through the CLN. Lymphatic drainage is inferiorly and to the nearby lymph nodes when tumors are located in the middle third.

US is the most routinely recommended primary preoperative evaluation for lymph node metastases in patients with PTC (8, 9). Metastatic lymph nodes tend to be large, round, cystic, hypoechoic, calcified, and hypervascularized with a loss of hilar architecture (23, 24). US has higher sensitivities and specificities in the lateral neck than in the central neck, but the false-positive rates of US should not be ignored (25, 26). As reported by Patron et al., occult metastasis may occur at LN-level II (14). In our study, 26.10% (136/521) of patients with clinical lymph node-negative (cN0) and positive in the central compartment (cN1a) had LN-level II metastasis on postoperative pathological examinations. This was primarily because, once CLNM presence was confirmed on intraoperative frozen section biopsy, the extent of LND was further expanded. Notably, accuracy is limited by the experience of clinicians, the function of instruments, and the heterogeneity of patients. Therefore, we believe it is inappropriate to decide whether to dissect LN-level II only based on the US-reported LN status preoperatively.

The thyroid gland is supplied by a rich vascular network. Some studies have revealed that there appears to be a trend toward more lymph nodes and distant metastasis and poor prognosis when the vascular invasion is present (27–29). Similarly, vascular invasion is an independent risk factor for LLNM (30, 31). Our study demonstrated a significantly increased risk of LN level II metastasis when the vascular invasion was present (OR=6.759), as shown in the nomogram, with a high predictive value, and dissection of LN level II was necessary.

Most studies suggest that CLNM is associated with LLNM, whereas LLNM is associated with LN-level II metastasis (32–34). Thus, we calculated the correlation between the rate in each compartment and LN-level II metastasis. Koo et al. studied 52 patients with cN1b but no suspicions of metastasis before the operation and found that occult LN-level II metastasis was associated with positive LN-levels III and IV simultaneously (32). In contrast, their results showed that if preoperative US did not indicate LN-level III metastasis, LN-level II was considered negative. In our study, LN-level II was positive even though LN-level III was negative. The possible reasons for this anomaly are as follows. First, we included patients with cN1b that did not partition the lateral compartment; therefore, there were patients with preoperative LN-level II positivity. Second, the sample size was large. Nevertheless, it is undeniable that LN levels III and IV metastases are significantly correlated with LN-level II positivity.

This study demonstrated that the prognosis in patients with LN-level II metastasis was worse than in those who were LN-level II negative. Several studies have shown that lymph node metastasis is associated with an increased risk of recurrence and reoperation rate (5, 35). Recurrence may not necessarily affect mortality but may bring great psychological pressure to the patient. In addition, the risk of reoperation has increased. Therefore, it is necessary to identify the risk factors for LN-level II metastasis and formulate an appropriate surgical approach during the first operation.

We established a predictive model for LN-level II metastasis, based on the above risk factors and their effects on prognosis, with a C-index of 0.815 and 0.804 by bootstrapping validation; further demonstrating a high discriminative ability to guide clinicians. The limitation of the model is the lack of external center validation to evaluate the prediction model’s accuracy.

We have gone through extensive dissection and rough zoning for precise assessment and selective cleaning. In this process, we continue to summarize and strive for more precise dissection to minimize recurrence and complications.

This study had several limitations. Several clinicopathological features, such as the histological subtype and *BRAF* mutation, were not identified. This was a single-center retrospective study with a lack of prospective validation. Furthermore, we are currently examining more detailed pathological reports and follow-up data.

Our study found that the largest tumor diameter >20 mm, located in the upper pole, with cN1 and vascular invasion. The rates of CLNM, LN-levels III, and IV metastases were independent of LN-level II metastatic predictors. Subsequently, we developed a prediction model to better guide lymph node dissection in patients with PTC. LN-level II dissection should be individualized according to preoperative and intraoperative clinicopathological data.

## Data availability statement

The original contributions presented in the study are included in the article/supplementary material. Further inquiries can be directed to the corresponding author.

## Ethics statement

The studies involving human participants were reviewed and approved by The Ethics Committee of the First Affiliated Hospital of Chongqing Medical University. Written informed consent for participation was not required for this study in accordance with the national legislation and the institutional requirements.

## Author contributions

All authors made substantial contributions to the conception or design of the work. Material preparation, data collection, and analysis were performed by CH, YZ. The first draft of the manuscript was written by CH, XS and DH revised it critically for important intellectual content. All author contributed to the article and approved the submitted version.
